# Friendship as a social mechanism influencing body mass index (BMI) among emerging adults

**DOI:** 10.1371/journal.pone.0208894

**Published:** 2018-12-18

**Authors:** Meg Bruening, Irene van Woerden, David R. Schaefer, Daniel Hruschka, Alexandra Brewis, Corrie M. Whisner, Genevieve F. Dunton, Michael Todd, Punam Ohri-Vachaspati, Melissa N. Laska

**Affiliations:** 1 College of Health Solutions, Arizona State University, Phoenix, AZ, United States of America; 2 Department of Sociology, University of California–Irvine, Irvine, CA, United States of America; 3 School of Human Evolution and Social Change, Arizona State University, Tempe, AZ, United States of America; 4 Institute for Health Promotion & Disease Prevention, University of Southern California, Los Angeles, California, United States of America; 5 College of Nursing and Health Innovation, Arizona State University, Phoenix, AZ, United States of America; 6 Division of Epidemiology and Community Health, University of Minnesota, Minneapolis, MN, United States of America; University of Tennessee Health Science Center, UNITED STATES

## Abstract

**Background:**

Social relationships have been proposed as a significant factor shaping obesity risk. The first year of college, a period of major social, behavioral, and weight changes, provides a context well-suited to tracking longitudinally the impact of shifting friendships on weight outcomes. This study sought to identify social mechanisms impacting BMI change among emerging adults.

**Methods:**

An analytic sample of 276 college students (71.0% female, 52.2% non-White) provided repeated reports of relationships and BMI was measured up to four times during 2015–2016. Stochastic actor-oriented models were used to examine change in BMI through social influence and change in friendships over time, controlling for sex and race/ethnicity.

**Results:**

At baseline, mean BMI was 24.2±4.5 kg/m^2^. Overall, mean BMI increased over time; individual decreases in BMI were uncommon. There was a selection effect of BMI: participants with BMIs between 22 and 26 kg/m^2^ were most likely to be nominated as a friend. While participants did not select friends based on BMI similarity, participants who were reported as friends were more likely to experience convergence in BMI over time relative to the BMIs of non-friends (p = 0.015). An increase in BMI (versus stability or a decrease) was more likely for those whose friends had a higher BMI on average compared to participants whose friends had the same or lower BMI (OR = 2.85, 95% CI = 1.22, 6.71).

**Conclusion:**

Analyses indicated BMI affected friend selection, not through students selecting friends with similar BMI, but rather, by students avoiding friends with more extreme BMI levels.

## Introduction

Current models of the etiology of weight gain strongly favor individual and community-level factors, creating a heavy focus on dietary and exercise decisions [1–5] and proximate food environments [6, 7] as the drivers of obesity. Limited emerging evidence indicates that inter-personal factors such as friendships may also have a critical influence on weight outcomes.

Social network analysis (SNA) is an especially effective means of quantifying how health behaviors and outcomes are shared, transferred, and influenced through the formation and maintenance of social ties [8]. Christakis and Fowler investigated the effects of friendship networks among participants in the longitudinal Framingham Heart Study and reported that obesity, and a range of other public heath-relevant factors like happiness, loneliness, and smoking behaviors, “spread” like infectious diseases [9–12]. However, their secondary analyses could not rigorously address potential causal mechanisms. Weight-related attitudes and behaviors among friendship groups do predict body dissatisfaction [13, 14], dieting onset [15], chronic dieting [16], unhealthy weight control behaviors [13–15], and symptoms of eating disorders [13]. Among adolescents, friends’ behaviors have been shown to be associated with both healthy and unhealthy eating, physical activity, and sedentary behaviors [3–5]. Friend influence has been associated with the adoption of other risky health behaviors such as smoking, binge drinking, and early sexual initiation [17]. In a simulation study, Zhang et al. tested how physical activity changed among a network of elementary students at two afterschool programs. Results suggested that using social network data to target popular children can result in positive changes in activity levels.

Subsequent studies have made an effort to better describe the underlying mechanisms that link friendship and weight gain [4, 18, 19]. A recent review synthesized the limited SNA literature examining friendship networks and obesity [20]. Out of 4 longitudinal studies included in the review, three reported that friendship influence resulted in higher body weights over time [21–23]; the fourth found null results related to influence, finding instead that similarity in weight among friends was attributable to friend selection [24]. Friendship appears to matter [4, 20], but the lack of fine-grained longitudinal datasets hinders attempts to infer cause-and-effect relationships, particularly among populations of youth and critical stages in the life course when there are rapid changes in health behaviors and friendships (e.g., toddlerhood, adolescence, emerging adulthood, older adulthood).

Longitudinal studies with several time points over short intervals are needed to better understand the “spread” of obesity across friendship networks, and may also better identify critical time points that can best be targeted by interventions [9, 21, 24]. Assessments that are more frequent may help us to better identify critical time points that can best be targeted by interventions. The equivocal state of the current evidence may help explain why the role of friendship in weight gain is so poorly integrated into these traditional models of obesity etiology [25]. More research among populations experiencing rapid weight gain concurrent with significant developmental transitions (i.e., emerging adulthood) is needed to better understand the role that friendships play in contributing excess weight gain.

Using a longitudinal design, this study investigated dynamic interactions occurring in friendship networks over time. To identify mechanisms (selection, influence, or both) by which friends impact BMI change among a diverse group of young adults, we applied social network analysis. We studied college students living in residence halls for three reasons. First, college students are at particular risk for weight gain, which ultimately tracks into adulthood [26, 27]. This period of “emerging adulthood,” the time after adolescence and before adulthood, is a time of important physical, social, and behavioral changes. The start of college life in particular can amplify social and behavioral changes during this stage of life when many students first move away from family households [28]. College students generally have very poor diets and low levels of physical activity, both of which have been associated with increased risk of overweight/obesity on into adulthood [27, 29, 30]. Second, college is also a time of new and rapidly shifting friendships. Social development and growth of complex friend relationships are in fact hallmarks of emerging adulthood [31, 32]. As youth transition to adulthood, they become more involved and intimate with friends, increasingly sharing thoughts and feelings and supporting expansion of personal beliefs and ideas [32, 33].Understanding peer effects during this important transition—a time when independence from the family and home environment and at a time when risk for weight gain, unhealthy diet, and low levels of activity is high—could provide unique insights for network-based interventions in emerging adult populations. Third, the co-resident setting served to concentrate social activity and made it more feasible to identify and then track salient friendships. Given the previous literature, we hypothesized that we would observe both an influence and selection effect on BMI among friendship networks of emerging adults.

## Methods

The data used for this study are derived from SPARC (Social Impact of Physical Activity and Nutrition in College). Details of the study methods can be found elsewhere [34]. The SPARC study included 1435 participants from 6 residence halls at a large public university. Participants completed web-based surveys via Qualtrics and trained research assistants measured their anthropometrics at the start and end of the Fall 2015 (Time 1 [T1] & Time 2 [T2]) and Spring 2016 (Time 3 [T3] & Time 4 [T4]) semesters. All participants provided written consent and all study protocols were approved by the Arizona State University Institutional Review Board.

### Friendship network sample

Our analytic method involves comparing the influence of friends to the influence of peers who could have been friends but were not. The most practical means to gather data on individuals and relevant friends and peers is to focus on a relatively bounded context, in which individuals have relationships that are salient to the outcome at hand. Such an approach necessarily ignores relationships outside the context. Thus, key to the suitability of this approach is identifying a setting that meets these criteria. Participants were recruited from residence halls because such settings concentrate friendship activity through students living together and, because halls are tied to majors, taking classes together. The percentage of students in the residence halls who participated in the study ranged from 28%-70%. Only participants from the residence hall with the highest compliance rates were included in this analysis, as low compliance from a given location can bias parameter estimates and decrease precision in the social network model [35]. Additionally, only students whose anthropometrics were measured by trained research staff on at least two occasions were included in this analysis. As such, the analytical sample for this study included a total of 276 students (42%) from the residence hall with highest compliance rates, and 19% of the overall sample.

Sensitivity analyses were conducted to examine model fit when dorms with lower saturation rates were included. Coefficients did not differ meaningfully in these models, but standard errors increased. This indicated that either we lost power when including residence halls with higher rates of non-participation (rates that exceeded recommendations [35]), or captured increased variability in modeled effects across contexts, though with a similar average effect. While the majority of students living in the residence halls were first-year students, some non-first-year students were also present. As the social networks of the first-year and non-first-year students were integrated, both types of students were included in the analysis. No difference between the analytical sample and the overall sample for the residence hall was observed for BMI (p = 0.904) and race/ethnicity (p = 0.348) reported at baseline, however the analytical sample had significantly more females (p = 0.008) and non- first-year students (p = 0.041) than the overall sample.

### Measurements

#### Body mass index

Portable Seca flat scales and Seca stadiometers were used to obtain weight and height measurements. Measurements were recorded by trained research staff to the nearest 0.1 kg/cm respectively. Body Mass Index (BMI) was calculated as kg/m^2^. BMI was grouped into 11 categories as the software used for social network analyses (RSiena) does not yet accommodate outcomes measured as continuous variables. BMI values between 19 and 25.99 were truncated and treated as integers, BMI values less than 19, between 26 and 27.99, between 28 and 30.99, and greater than or equal to 31 were categorized into four additional categories respectively. Our analysis software required that these BMI groupings be mean centered, though for readers’ interpretation, we report in non-centered units.

#### Friendships

At each phase, participants were asked to "Please rank your top 5 male and top 5 female friends at ASU (the first being your best friend, the second being your next closest friend, and so on)." Friendship ties between study participants were coded as either present or absent and treated as being directed *from* the participant *to* the nominated friend. If a participant nominated a person who was also included in the study, the (directed) friendship tie *from* the participant *to* the nominated friend was coded as present. This tie was measured separately from the tie *from* the nominated friend *to* the participant. If a participant did not did not nominate another participant as a friend, that tie was coded as absent.

#### Individual attributes

Information on participants’ race/ethnicity, sex, year in college, and place of residence were collected. Race/ethnicity was determined by asking "How do you usually describe yourself? (check all that apply)", with response options White, Black or African American, Hispanic or Latino/a, Asian or Pacific Islander, American Indian or Alaska Native, some other race (please specify). Based on distributions, race/ethnicity was coded as Hispanic, non-Hispanic White, non-Hispanic Black, or Other for all analyses. Given the small number of non-first-year students, year in college was coded as first-year vs. non-first-year student.

### Data analysis

Stochastic actor-oriented models (SAOMs) were used to examine change in friendship ties and BMI over time [36]. As participant demographics could potentially be used to identify the participants, only the dataset without demographics is included for readers. To ensure participant confidentiality, additional permissions are needed to share the dataset with participant demographics. To permit readers to reproduce and compare analyses with the available data excluding demographics, we report analyses with the non-demographic data in addition to the full analyses. Detecting peer influence among friends is challenging because without random assignment to relationships, selection into friendships remains a threat to inference. A strength of the SAOM is that it addresses selection into friendships by explicitly modeling network change over time. Separate functions in the SAOM were used to estimate network change (whether a friendship tie will form, persist, or terminate) and BMI change (increase, stability, or decrease); parameters in both functions were estimated simultaneously. SAOMs assume the observed data are snapshots of a continuous process and hence estimable as a Markov process [37]. The model assumes change occurs through a series of unobserved ministeps, interspersed between observation waves, with only one actor able to make one change (in outgoing ties or BMI) during a single ministep. All variables in the SAOM were mean centered. For instance, the 11 BMI groupings, which initially coded with integer values from 1 to 11, were centered around the mean value, resulting in a range from -5.25 to 4.75.

The network function models selection into friendships, i.e., a prediction equation in which the outcome is whether or not a tie is present or absent in a given dyad (between two participants). This function was specified using student demographics, residence location, BMI, and a set of common network processes (e.g., reciprocity, transitivity) as predictors. Three types of effects related to BMI were included to determine if BMI was associated with friendship nominations. An *ego* effect tested whether participants with higher BMI were more, or less, likely to nominate someone as a friend. An *alter* effect tested whether participants with higher BMI were more, or less, likely to be nominated by someone else as a friend. To allow for nonlinearities in these forms of selection, both a linear and quadratic term for each effect was specified. An interaction of the ego and alter BMI was also specified to determine if participants preferred friends who had BMI similar to their own.

The network function included controls for several other factors that shape friend selection. Ego and alter effects for sex, race/ethnicity, and year in college were included to evaluate how these attributes affected the likelihood of nominating a friend or being nominated as a friend. Sex, race/ethnicity, year in college, and residence location were also examined to determine if participants were more likely to nominate a friend who had the same value on an attribute versus someone different.

Models included endogenous network effects to account for typical network structures and processes that drive friendship change. These included an outdegree effect (a control for the overall probability of a friendship tie), reciprocity (if someone nominated the participant as a friend and the participant also nominated that person), transitivity (whether naming a friend’s friend as one’s own friend has a higher probability), and the interaction of reciprocity with transitivity. In addition, indegree activity and outdegree activity effects controlled for whether participants were more likely to have nominations/be nominated as a friend if they already had a higher number of incoming and outgoing ties, respectively.

The behavior function models participants’ change in BMI over time. Two effects were used to represent the distribution of BMI. In combination, linear and quadratic terms of BMI were used to represent the tendency for participants to adopt particular values of BMI based on their current value (e.g., whether there is a central tendency or individuals tend toward extremes). To examine if participants tended to have BMI changes in the direction of their friends BMI, the average similarity effect was used. This tests if a participant’s BMI changes over time to become more similar to the average of their friends’ BMI values. Finally, covariates were included to represent the effects of participant sex, race/ethnicity, and year in college on BMI. Interpretation of the behavior function results was facilitated by calculating the predicted likelihood of adopting different BMI values based on the BMI of oneself and one’s friends during a model ministep. A Monte Carlo bootstrap approach was used to determine 95% CIs.

An unrestricted model allowing for time heterogeneity was examined. The model effects with and without time heterogeneity were not significantly different; as such, the most parsimonious model is presented. Non-significant network and behavior function demographic effects were also removed from the SAOM for parsimony. The data and RSiena code for this de-identified analysis is available as a supplement. All analyses were conducting using the statistical software R (version 3.3.3) and package RSiena (version 1.1–302). In order to assist in interpretability of findings in the text of the results section, β values from the tables were converted to odds ratios. Statistical significance was determined at p<0.05.

## Results

**Fig 1:**
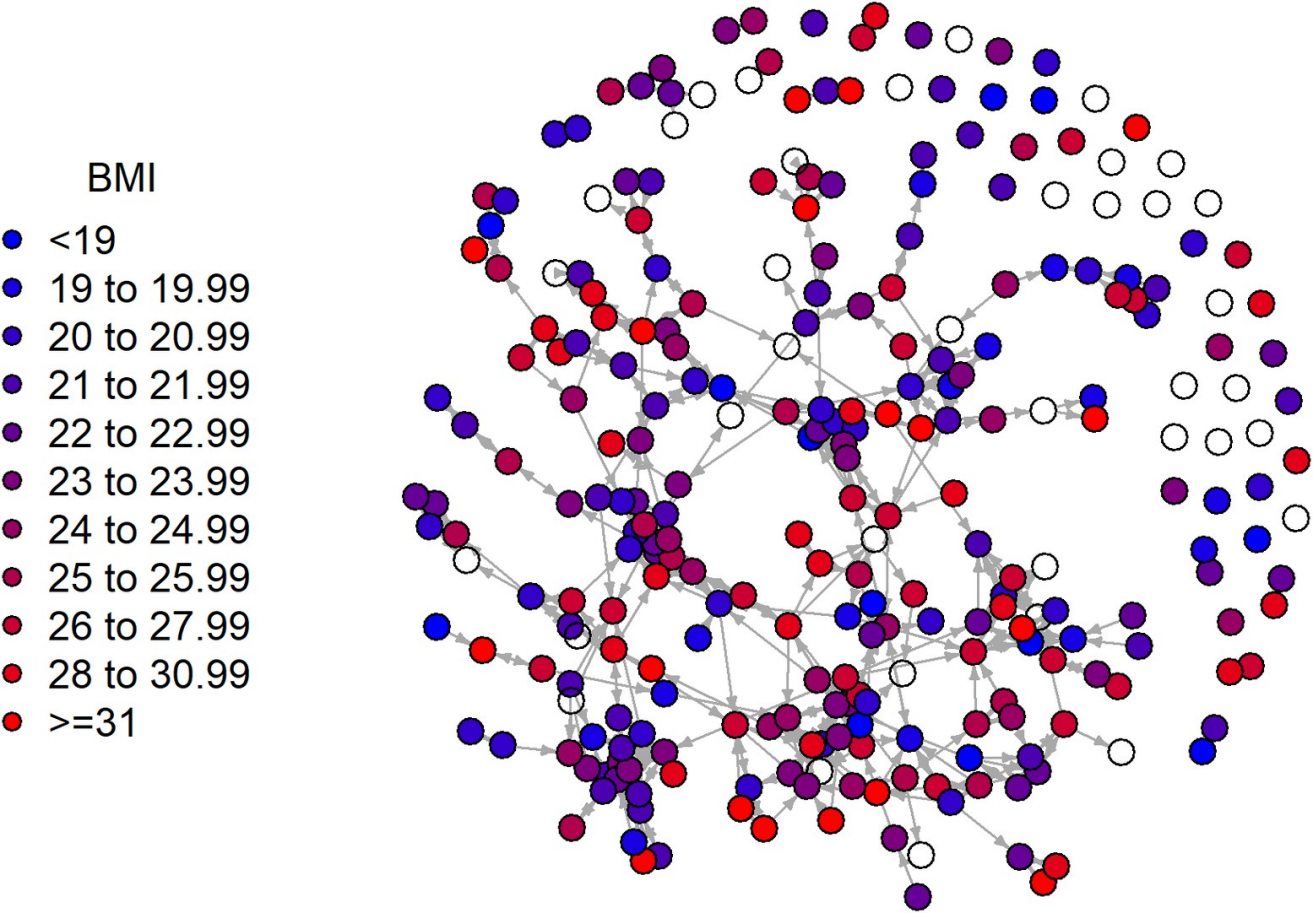
The social network and BMI category at Time 1 among participants of emerging adults (n = 276).

A total of 276 participants who had at least two time points of data were included in analyses (71.0% female, 52.2% non-White, 93.1% first-year students) (See Table 1 for descriptive characteristics of those included at each time point). Of the 276 participants, 73% provided information for at least three time points. As information was obtained at the start and end of each semester, these participants provided information in both semesters. Of the 75 participants who only provided information at two time points, 38 participants only provided information in the Fall semester and 13 participants only provided information in the Spring semester. At T1, mean BMI was 24.2±4.5 kg/m^2^. Between T1 and T4, 40% of participants increased their BMI category as defined in the methods section; 46% of participants remained in the same BMI category, and 14% decreased their BMI category. The number of friendships reported among the participants decreased over time. Participants nominated an average of 8.0 friends at T1, and an average of 6.8 friends at T4. The number of friendships reported within the residence hall also decreased over time, from an average of 3.3 ± 2.0 ties at T1 to 2.8 ± 1.8 at T4. See Fig 1 for a visual summary of the network.

**Table 1:** Descriptive statistics of the analytic sample of the social network of emerging adults at each time point during the 2015–2016 academic year.

		Time 1	Time 2	Time 3	Time 4
		(n = 239)	(n = 241)	(n = 218)	(n = 192)
Sex, n (%)					
	Female	172 (72.0)	170 (70.5)	160 (73.4)	134 (69.8)
	Male	67 (28.0)	71 (29.5)	58 (26.6)	58 (30.2)
Race/ethnicity, n (%)					
	Black	25 (10.5)	25 (10.4)	23 (10.6)	21 (10.9)
	Hispanic	65 (27.2)	67 (27.8)	60 (27.5)	57 (29.7)
	Other	32 (13.4)	31 (12.9)	31 (14.2)	28 (14.6)
	White	117 (49.0)	118 (49.0)	104 (47.7)	86 (44.8)
Year in college, n (%)					
	First-year student	221 (92.5)	223 (92.5)	200 (91.7)	178 (92.7)
	Non-first-year student	18 (7.5)	18 (7.5)	18 (8.3)	14 (7.3)
BMI, mean (SD)		24.23 (4.52)	24.69 (4.53)	24.47 (4.63)	24.96 (5.02)
BMI category, n (%)					
	<19	12 (5.0)	12 (5.0)	10 (4.6)	6 (3.1)
	19 to 19.99	15 (6.3)	14 (5.8)	9 (4.1)	11 (5.7)
	20 to 20.99	31 (13.0)	23 (9.5)	30 (13.8)	25 (13.0)
	21 to 21.99	34 (14.2)	33 (13.7)	34 (15.6)	22 (11.5)
	22 to 22.99	22 (9.2)	18 (7.5)	12 (5.5)	11 (5.7)
	23 to 23.99	24 (10.0)	25 (10.4)	27 (12.4)	17 (8.9)
	24 to 24.99	16 (6.7)	22 (9.1)	19 (8.7)	27 (14.1)
	25 to 25.99	18 (7.5)	18 (7.5)	9 (4.1)	18 (9.4)
	26 to 27.99	28 (11.7)	27 (11.2)	23 (10.6)	13 (6.8)
	28 to 30.99	21 (8.8)	30 (12.4)	27 (12.4)	22 (11.5)
	≥31	18 (7.5)	19 (7.9)	18 (8.3)	20 (10.4)

### Network function results

When the model excluded demographics, the network function results indicated that BMI affected friend selection. The squared BMI alter effect was (β = -0.021, p = 0.009), indicating that participants were most likely to be nominated as a friend if their BMI was between 22 and 26 kg/m^2^ (Table 2). For the model including demographics, we begin by interpreting the effects for the controls to help convey the intuition behind the SAOM. A significant male alter effect was found (i.e., compared to females, males were more likely to be nominated as a friend by another participant, OR = 1.6, 95% CI = (1.3, 1.9); β = 0.441, p<0.001) (Table 3). There were multiple race/ethnicity effects. To determine the overall effect of race/ethnicity, all effects related to race/ethnicity must be considered jointly. The effects were a White alter effect (compared to non-Whites, Whites were less likely to be nominated as friends, OR = 0.8, 95% CI = (0.7, 1.0); β = -0.228, p = 0.015) and a Hispanic ego effect (Hispanics were less likely than non-Hispanics to nominate friends, OR = 0.8, 95% CI = 0.6, 1.0); β = -0.266, p = 0.046) were observed. In addition, a similarity effect for Whites was found, indicating that nominations between White participants, and nominations between non-White participants, were more likely than nominations between White and non-White participants (OR = 1.3, 95% CI = (1.1, 1.5); β = 0.224, p = 0.006). The combination of these effects indicate that overall, White participants were as likely to nominate friends who were White versus other races, whereas non-Whites were more likely to have non-White, versus White, friends. Specifically, compared to White participants, Hispanics were less likely to name a White friend (OR = 0.6) and just as likely to name a non-White friend (OR = 0.96), while other non-White participants were also less likely to name a White friend (OR = 0.80), but more likely to name a non-White friend (OR = 1.26).

**Table 2:** Estimates from stochastic actor-oriented models on the association between ego and alter^a^ BMI and friendship networks when demographics excluded from the analysis (n = 276).

**Network functions (Friendship change)**	β	Std. Error	P value
Rate (period 1) ^1^	4.250	0.344	<0.001
Rate (period 2) ^1^	2.221	0.197	<0.001
Rate (period 3) ^1^	3.310	0.274	<0.001
Outdegree (density) ^2^	-2.341	0.211	<0.001
Reciprocity ^2^	4.248	0.242	<0.001
Transitive triplets ^2^	1.002	0.092	<0.001
Transitive reciprocated triplets ^2^	-0.666	0.125	<0.001
Outdegree–activity ^2^	-0.149	0.042	<0.001
Indegree–activity ^2^	-0.221	0.077	0.004
BMI alter ^3^	0.021	0.018	0.246
BMI alter squared ^3^	-0.021	0.008	0.009
BMI ego ^4^	0.003	0.020	0.871
BMI ego squared ^4^	0.002	0.009	0.866
BMI ego x BMI alter ^5^	0.005	0.005	0.348
**Behavior functions (BMI change)**	β	Std. error	P value
Rate (period 1) ^1^	1.059	0.124	<0.001
Rate (period 2) ^1^	0.738	0.098	<0.001
Rate (period 3) ^1^	0.892	0.120	<0.001
BMI linear shape ^6^	0.310	0.068	<0.001
BMI quadratic shape ^6^	0.041	0.023	0.072
BMI average similarity (influence) ^7^	5.205	2.239	0.020

^a^The participant is an ego. A participant’s friend is an alter.

^1^Rate indicates the number of opportunities for (friendship or BMI) change.

^2^ Outdegree (density), reciprocity, transitive triplets, transitive reciprocated triplets, outdegree–activity, indegree–activity are all controls.

^3^ BMI alter and BMI alter squared examine if the friend’s BMI is associated with friendship selection.

^4^ BMI ego and BMI ego squared examine if the participant’s BMI is associated with friendship selection.

^5^ BMI ego x BMI alter is an interaction effect.

^6^ BMI linear shape and BMI quadratic shape indicate which BMI values were likely to be adopted

^7^ BMI similarity examines if participants’ BMI tends to become more similar to the average of their friends' BMI

**Table 3:** Estimates from stochastic actor-oriented models on the association between ego and alter^a^ BMI and friendship networks (n = 276).

**Network functions (Friendship change)**	β	Std. Error	P value
Rate (period 1) ^1^	4.399	0.378	<0.001
Rate (period 2) ^1^	2.248	0.215	<0.001
Rate (period 3) ^1^	3.406	0.325	<0.001
Outdegree (density) ^2^	-3.779	0.277	<0.001
Reciprocity ^2^	4.197	0.263	<0.001
Transitive triplets ^2^	0.973	0.091	<0.001
Transitive reciprocated triplets ^2^	-0.681	0.119	<0.001
Outdegree–activity ^2^	-0.157	0.044	<0.001
Indegree–activity ^2^	-0.209	0.075	0.005
Male alter ^3^^b^	0.441	0.098	<0.001
White alter ^3^	-0.228	0.094	0.015
First year student alter ^3^	-1.033	0.172	<0.001
Hispanic ego ^4^	-0.266	0.133	0.046
Same floor ^5^	0.418	0.105	<0.001
Same White ^5^	0.224	0.081	0.006
Same year in college^5^	1.295	0.191	<0.001
BMI alter ^6^	0.014	0.019	0.465
BMI alter squared ^6^	-0.020	0.008	0.012
BMI ego ^7^	-0.002	0.020	0.913
BMI ego squared ^7^	-0.003	0.009	0.739
BMI ego x BMI alter ^8^	0.006	0.006	0.319
**Behavior functions (BMI change)**	β	Std. error	P value
Rate (period 1) ^1^	1.061	0.120	<0.001
Rate (period 2) ^1^	0.736	0.099	<0.001
Rate (period 3) ^1^	0.893	0.118	<0.001
BMI linear shape ^9^	0.311	0.067	<0.001
BMI quadratic shape ^9^	0.041	0.022	0.065
BMI average similarity (influence) ^10^	5.233	2.144	0.015

^a^The participant is an ego. A participant’s friend is an alter.

^b^The non-significant network and behavior function demographic effects were removed from the SAOM for parsimony.

^1^Rate indicates the number of opportunities for (friendship or BMI) change.

^2^ Outdegree (density), reciprocity, transitive triplets, transitive reciprocated triplets, outdegree–activity, indegree–activity are all controls.

^3^ Male alter, White alter, and first year student alter examine if participants who are male, White, and first year students are more likely to be nominated than female, non-White, and non-first year student participants.

^4^ Hispanic ego examines if participants who were Hispanic were more likely to nominate friends than non-Hispanic participants.

^5^ Same floor, same White, and same year in college examine if participants living on the same floor, or with the same White/year in college attribute are more likely to nominate each other as friends.

^6^ BMI alter and BMI alter squared examine if the friend’s BMI is associated with friendship selection.

^7^ BMI ego and BMI ego squared examine if the participant’s BMI is associated with friendship selection.

^8^ BMI ego x BMI alter is an interaction effect.

^9^ BMI linear shape and BMI quadratic shape indicate which BMI values were likely to be adopted

^10^ BMI similarity examines if participants’ BMI tends to become more similar to the average of their friends' BMI

An alter effect for year in college (OR = 0.4, 95% CI = 0.3, 0.5; β = -1.033, p<0.001) and a similarity effect on year in college (OR = 3.7, 95% CI = 2.5, 5.3; β = 1.295, p<0.001) were observed, indicating that participants were more likely to nominate each other if they were both first-year students, or both non-first-year students; an ego-alter selection table [37] indicated that the effect was weaker among first year student participants. The significant effect for participants living on the same floor of the residence hall (β = 0.418, p<0.001) indicates that participants were 1.5 (95% CI = 1.2, 1.9) times more likely to nominate each other if they were living on the same floor versus different floors.

**Fig 2:**
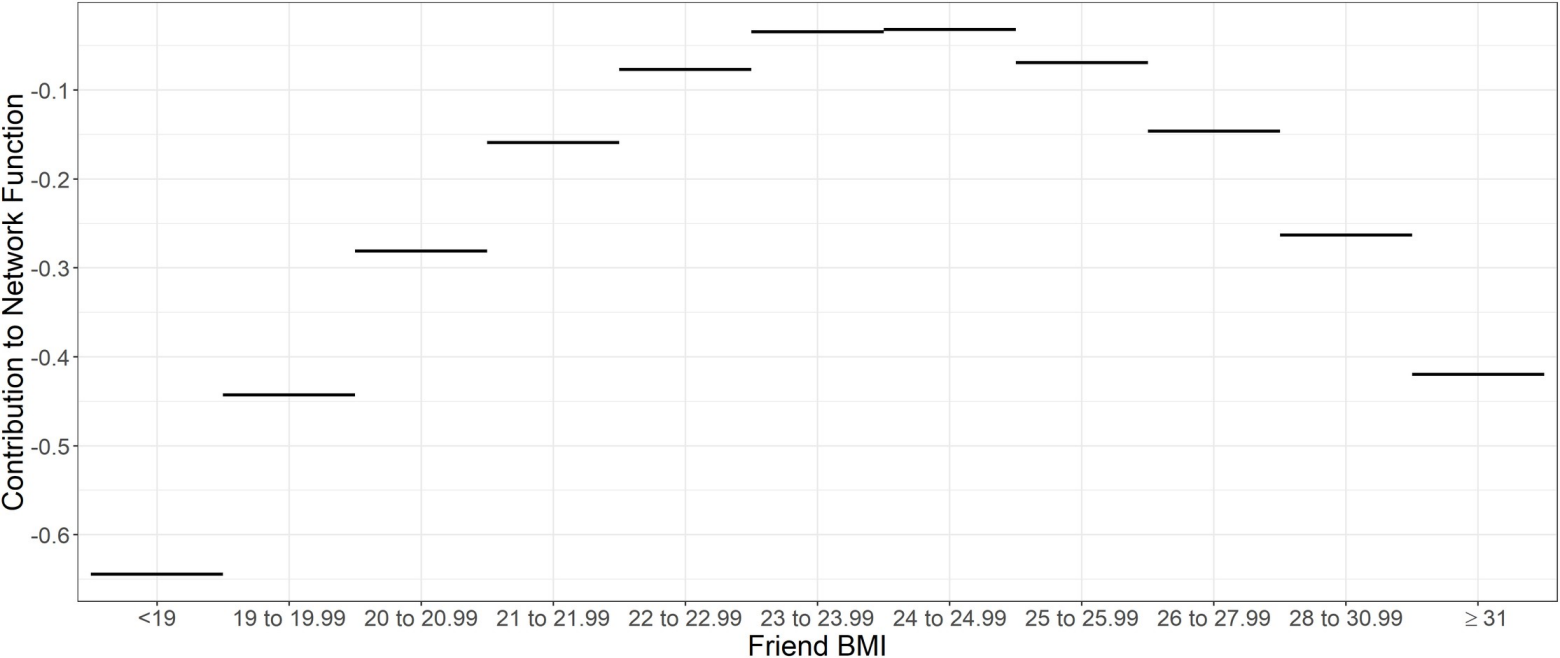
Predicted effect of BMI on the tendency to be nominated as a friend.

Results also indicate that BMI affected friend selection. The squared BMI alter effect was negative (β = -0.020, p = 0.012). Because BMI was centered prior to forming the quadratic term (i.e., low BMIs had negative values and high BMIs had positive values, both yielding positive squared BMI values), this finding indicates a lower likelihood of nominating alters whose BMI was further from the average BMI (where squared BMI = 0). Participants were most likely to be nominated as a friend if their BMI was between 22 and 26 kg/m^2^ and the least likely to be nominated as a friend if their BMI was less than 19 and greater than 26 (Fig 2). No other significant individual attributes (e.g., no effect was observed for participants preferentially nominating others of the same sex, or for participants nominating White participants) or BMI effects (e.g., BMI alter, BMI ego, BMI ego x BMI alter) were found.

### BMI function results

The linear and quadratic shape effects indicate the probability of being in each BMI category. When participant demographics were excluded from the model, the BMI linear shape (β = 0.310, p<0.001) and BMI quadratic shape (β = 0.041, p = 0.072) indicated that participants were more likely to be in the higher BMI categories than the lower BMI categories, and a possible bimodal distribution (Table 2). In models with participant demographics, the linear shape parameter was positive and significant (Table 3:β = 0.311, p<0.001), indicating participants were more likely to be in the higher BMI categories than in the lower BMI categories. The positive quadratic shape parameter suggested a bimodal distribution of predicted probabilities across the ordered BMI categories, such that probabilities were higher toward or at the high and low extremes of BMI, as compared to the middle categories. This parameter, however, was not significant (β = 0.041, p = 0.065). Overall, the linear and quadratic shape effects indicate a tendency toward higher levels of BMI.

In terms of peer influence on BMI, in models without demographics, the BMI average similarity (Table 2: β = 5.205, p = 0.020) indicated participants’ BMI changed to become more like their friends’ BMI over time. The odds ratios and 95% CI calculated from the BMI function results changed slightly: participants whose friends had a higher BMI were significantly more likely to increase a BMI category (OR = 2.83, 95% CI = 1.17, 6.93); a decrease in BMI for these participants was significantly unlikely (OR = 0.35, 95% CI = 0.14, 0.85). In models adjusted for demographics, the BMI average similarity effect was also statistically significant (Table 3: β = 5.233, p = 0.015), indicating participants’ BMI changed to become more like their friends’ BMI over time. Compared to participants whose friends had the same or a lower BMI, participants whose friends had a higher BMI were significantly more likely to increase a BMI category (OR = 2.85, 95% CI = 1.22, 6.71); a decrease in BMI for these participants was significantly unlikely, as indicated by the inverse of the previous estimate (OR = 0.35, 95% CI = 0.15, 0.82). Compared to when a participant had the same BMI as their friends, the odds of increasing BMI were 9% higher for each increase in the participants BMI category (OR = 1.09, 95% CI = 0.49, 2.43), although this was not statistically significant. Participants’ sex, race/ethnicity, and year in college were not significant predictors of BMI change over time.

## Discussion

From a life course perspective, college students are at a critical transition, in which they are newly independent leaving family environments and have new autonomy over health choices and tend to experience heightened social (i.e., friendships) and physical (i.e., excess weight gain) changes. We sought to study the unique population of emerging adults in college because of the possibility of excess weight gain during this period impacting their long-term health. This study examined how changes in college students’ BMI during an academic year might be explained by their friendship networks. We used a longitudinal social network methodology that allowed us to estimate the strength of friend influence on BMI while simultaneously controlling for the effect of BMI on selection into friendship. Using data from four time points, we observed a significant effect of friends’ BMI on increasing BMI among the students in our sample. In addition, while students were more likely to be befriended if their BMI was between 22 and 26, we did not otherwise observe selection based upon BMI similarity. These findings can be used to test and shape interventions to prevent excess weight gain during college.

Research has repeatedly shown that overweight youth are less likely to be nominated as friends by others [38, 39], and youth of similar weights tend to cluster, suggesting a possible selection effect [40]. Contrary to much of the research in adolescence [24, 41], we did not observe students sorting into friendships based on BMI similarity. This suggests that at this post-adolescence stage of development, similarity in weight status may not be as important as other factors in promoting friendship [42]. Interestingly, we did observe that BMI affected friend selection, not through students selecting friends with similar BMI, but rather, by students avoiding friends with more extreme BMI levels. This finding is troubling as students with extreme BMIs may be excluded and marginalized. These findings suggest work is needed to not only promote healthy bodies, but also to work with youth and friend groups, even among emerging adults, to decrease the stigma with being of a certain weight status, including those at lower weights.

Longitudinal research among middle school and high school adolescents in the US and Australia have indicated that friends’ BMI exerts influence on one’s own BMI over the course of two time points within 1 year and 5 year intervals, respectively [21, 24]. We observed a similar influence effect over 4 time points over 9 months, with participants almost 3 times more likely to have an increase in BMI if their friends had a higher BMI than their own, compared to if friends had the same or lower BMI than oneself. Our results suggest that friends' BMI does influence BMI progression over time. Given evidence of friend influence, it may be beneficial for interventions to work with groups of friends to encourage healthy behaviors to promote healthy weights or limit excess weight gain. Behavioral studies suggest that healthy and unhealthy eating and physical activity behaviors are similar among friends [3, 5]. Intervention studies have yet to show a difference in weight status as a result of friend/peer behavior change programs [43, 44], although promising results have been seen among married spouses [45] and simulation studies [46]. Future studies are needed to examine the mechanism through which the influence of friends’ BMI interacts with behavioral factors that result in increasing BMI among youth, so that interventions among friends can be better designed. This study provides a starting point for social network intentions among emerging adults.

### Strengths and limitations

Though diverse, the sample for this study was limited to college students from one large southwestern university. We objectively measured anthropometrics over several points in time over the course of one year for both the individual and the nominated friends. The longitudinal design of the study allows us to better disentangle the causal effects of selection and influence. Original plans for the study included sampling from multiple residence halls, but challenges in recruitment limited our ability to do so [34]. Thus, only one residence hall yielded adequate saturation for social network analyses. In addition, participants were asked to limit the nomination of friends to others at ASU. This approach may have limited other salient friendships that may have contributed to change in BMI.

This study is the first of its kind to examine how social networks impact changes in body mass index among diverse college students over the course of an academic year. Social network analyses indicated BMI affected friend selection, not through students selecting friends with similar BMI, but rather, by students avoiding friends with more extreme BMI levels. In addition, we observed that friends influence one another’s increases in BMI over time. This study does not control for the activities friends engaged in together, which may be associated with weight change. These findings point to the need for more research to understand the behavioral mechanisms by which friends influence weight gain.

## Supporting information

S1 File BMI.csv. Participants’ body mass index at each of the four time points (T1-T4).
(CSV)

S2 File Links_all.csv. Participant friendship links (ego and alter) nominations for each of the four time points (T1-T4).
(CSV)

S3 File RSienaScript—friendship as a social mechanism. The R code to conduct deidentified analyses of stochastic actor-oriented models on the association between ego and alter BMI and friendship networks.
(R) Click here for additional data file.
